# Evaluation of serological response to foot-and-mouth disease vaccination in BLV infected cows

**DOI:** 10.1186/s12917-016-0749-x

**Published:** 2016-06-21

**Authors:** Rodrigo Puentes, Laureana De Brun, Agustina Algorta, Valeria Da Silva, Florencia Mansilla, Gustavo Sacco, Silvia Llambí, Alejandra V. Capozzo

**Affiliations:** Immunology Area - Department of Microbiological Sciences, Faculty of Veterinary, University of the Republic (UdelaR), Montevideo, Uruguay; Genetics Area, Faculty of Veterinary, University of the Republic (UdelaR), Montevideo, Uruguay; Centre of Veterinary Sciences and Agronomic Investigations, INTA, Virology Institute, Buenos Aires, Argentina; Independent Veterinary Practice, Florida, Uruguay; CONICET – National Council of Scientific and Technological Research, Buenos Aires, Argentina

**Keywords:** Immune response, BLV, Serology, FMD, Immunization, Dairy cattle

## Abstract

**Background:**

Bovine Leukemia Virus (BLV) produces disorders on the immune system in naturally infected animals, which may counteract the development of immunity after vaccination. The aim of this study was to investigate whether healthy and BLV infected cattle elicited similar humoral responses after foot and mouth disease (FMD) immunization. In a field study, 35 Holstein heifers were selected based on their BLV serological status and immunized with a single dose of a commercial bivalent oil-based FMD vaccine. Serum samples were collected at 0, 15, 60, 165 and 300 days post vaccination (dpv).

**Results:**

Total anti-A24/Cruzeiro antibodies, IgM, IgG1, IgG2 titers and avidity index of specific antibodies were determined by ELISA. Although only marginally significant differences were found between groups in terms of total antibodies, anti-FMD IgM and IgG1 titers were significantly lower in heifers infected with BLV at the 15 dpv (*p* < 0.01). Animals that became infected during the study did not show differences to the BLV negative group.

**Conclusions:**

Cattle infected with BLV at the time of immunization may elicit a low-magnitude serological response to a commercial Foot-and-mouth disease vaccine.

## Background

Enzootic bovine leucosis is a contagious disease of cattle induced by an exogenous retrovirus, bovine leukemia virus (BLV). It is worldwide distributed and only 20 countries had been able to eradicate the disease. Approximately 60 % of infected animals do not display clinical signs of disease, and these animals are referred to as asymptomatic or aleukemic. Approximately 30–40 % of BLV carriers will develop a persistent lymphocytosis, while fewer than 5 % develop malignant lymphosarcoma [1].

BLV positive animals at early stages of infection develop a cellular response mediated mainly by T helper 1 lymphocytes (Th1) producing IL-2, IL-12 and IFN-γ. Disease progression, together with persistent lymphocytosis produces changes on the T-cells profile towards a Th2 response [2]. In this phase, there is a dramatic increase in the B-lymphocyte populations and a decrease in the percentages of both CD4+ and CD8+ T lymphocytes [3]. The altered cytokine production was suggested to be responsible for the suppressed mitogen-induced T lymphocyte proliferation in BLV-infected animals [3, 4].

Current literature discuses if these BLV-induced immune mechanisms have a detrimental impact on the ability of cattle to resist the progression of infectious disease [5–7]. Considering that BLV is endemic in many countries and approximately 60 % of the animals are asymptomatic, it is important to know how BLV infection interacts with the immunogenicity of those vaccines usually used in bovines. An early study reported a possible impairment of immune responses against rotavirus in BLV-positive animals [8]. Recently, Erskine et al. [1] verified that dairy cows that were infected with BLV had decreased antibody responses to J5 *E. coli* bacterin as compared to non-infected cows.

Foot and mouth disease (FMD) is a highly contagious acute vesicular viral disease that affects cloven-hoofed animals and is mainly controlled by vaccination. The circulation of FMD virus (FMDV) in susceptible livestock imposes severe restrictions on the movement and trade of animals and derived products, causing serious economic loss to the affected countries [9]. FMD is endemic in many parts of Asia, Africa, and South America, where vaccination of susceptible populations is widely used as a major control measure. Commercial formulations usually contain more than one virus strain, as immune responses induced by vaccination are strain-specific [10]. Protection is mediated by specific antibodies. IgG1 has been related with protection in vaccinated cattle [11–14] while IgM mediates protection in naïve-infected cattle [15]. Maintaining high levels of total antibodies against FMDV is paramount to prevent outbreaks, keeping the OIE free-with-vaccination status and thus, the international markets.

There is no information on how the application of FMD vaccine in BLV infected animals may interfere with the immune response against FMDV. Considering the critical role that T- and B-cell populations play in humoral immunity and the immune-modulation caused by BLV in cattle, the purpose of this study was to investigate whether BLV natural infection may counteract the serological response to FMD primo- vaccination.

## Methods

### Animals

Thirty-five animals 6 to 10 months old Heifers were selected from a herd of 73 heifers according to their BLV antibodies status measured by ELISA twice (4 weeks and 1 week) before vaccination against FMD. The animals had not received anti-FMDV vaccine until the beginning of the experiment. They received one dose of FMD vaccine throughout the study, corresponding to FMD vaccination campaign of February 2014. Animals were housed in the same farm situated in the Department of Florida-Uruguay.

### Vaccine

A commercial oil-adjuvanted (water-in-oil) vaccine against FMD was used in this study. This is an oil-adjuvanted vaccine that contains two inactivated FMDV strains: O1/Campos and A24/Cruzeiro, produced by a Paraguayan manufacturer. This vaccine was approved by the “Ministerio de Ganadería Agricultura y Pesca” (MGAP) according to the current national regulations of Uruguay.

### Experimental design

The selected heifers were divided into 2 groups: BLV seropositive (BLV+, *n* = 20) and BLV seronegative (BLV–, *n* = 10). There were 5 seronegative animals at the day of vaccination (Day 0) seroconverted throughout the study, they were considered in a third group: Seroconverted (SC). Furthermore, all seropositive animals were tested by hemogram in peripheral blood mononuclear cell (PBMC) to detect leukocytosis or lymphocytosis at the beginning of the experiment using the protocol described by Marshak et al. [16]. All the selected animals were negative against anti FMDV antibodies at 0 dpv (liquid phase blocking ELISA titers ≤ 1.5).

All animals received one dose of 3 mL of FMD vaccine applied subcutaneously in the left side of the neck, according to current regulation in Uruguay [17]. Serum samples (2 aliquots of 2 mL each per animal) obtained at 0, 15, 60, 165 and 300 dpv were stored at −20 °C for further serological assessments.

### BLV antibody detection by ELISA

A commercial kit was used for detection of BLV specific antibodies in bovine sera (IDEXX, REF P02110-10 LOT 4155 N, The Netherlands. Samples were processed according to manufacturer’s instructions and the reading was performed at 450 nm in a visible range spectrophotometer (Thermo Fisher Scientific Inc., USA). Two weak positive controls were used per plate and interpretation was made according to manufacturer’s protocol.

### Liquid phase blocking ELISA (LPBE)

Total anti-FMDV A24/Cruzeiro antibody responses were assessed in serum samples by LPBE performed as stated by the OIE Manual using a rabbit antiserum to capture inactivated whole 140S viral particles, and a guinea-pig antiserum as detector antibody, both of them strain-specific as described before [18]. Antibody titers were expressed as the reciprocal Log10 of serum dilutions giving the 50 % of the absorbance recorded in the virus control wells without serum.

### Single dilution avidity ELISA

Avidity assessment of specific antibodies was performed at 15 dpv as described before [12]. The Avidity Index (AI) was calculated as the percentage of residual activity of the serum sample after a 20 min urea washing step, relative to that of untreated sample: AI% = (OD sample with urea/OD sample without urea) × 100.

### Isotype ELISAs

Isotype ELISAs were performed as reported before [12, 18] using HRP-conjugated antibodies anti IgG1 (1:750), IgG2 (1:750) and IgM (1:500) (AbD Serotec, Oxford, UK). Briefly, 96 well flat bottom well plates (MICROLON®, Greiner Bio-One, Monroe, NC) were coated with 50 μl per well of a dilution that contained 15 ng/well of sucrose-gradient purified FMDV 146S particles of A24 Cruzeiro strain, and blocked with dilution buffer. Individual serum samples were run in two-fold serial dilutions starting at 1:50. Titers were expressed as the inverse dilution reaching the cut off value (0.2) calculated as mean OD + 2SD achieved by the FMDV-negative Patagonian bovine serum samples (*n* = 25). Titers were expressed as the dilution factor reaching the cut-off value [12].

### Data analysis

The “expected protection percentage” (EPP) was used as a reference to protective vaccine-induced responses. The EPP relates antibody titers measured by LPBE at 60 dpv, with the percentages of protection achieved for the same groups of animals after in vivo challenge experiments performed at 90 dpv following the “protection against generalized foot infection” (PGP) test. LPBE titers corresponding to EPP values = 75 % (EPP-75 %) are 1.90 for A24/Cruzeiro strain [19, 20].

Time-course titers obtained by LPBE, AI and IgG-subtype ELISAs were plotted and results between the three experimental groups were compared by ANOVA 2-factor repeated measures followed by Bonferroni multiple comparisons test. Mann-Whitney test was used when data from the groups were compared. The confidence interval was 95 %. Statistical analyses were carried out using GraphPad Prism v5.0 (GraphPad Software).

## Results

### Total antibodies against BLV and hemogram results

**Fig. 1:**
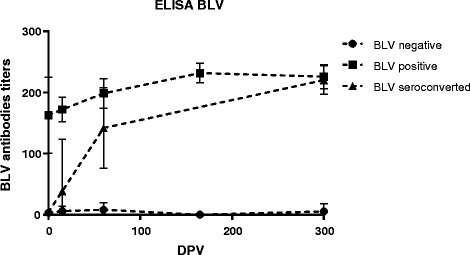
Kinetics of BLV Ab titers of each group of animals throughout the study measured by indirect ELISA. Mean titers standard errors (SEM) are depicted. BLV – and BLV+ animals were negative and positive respectively throughout the experiment. The “BLV seroconverted” group corresponds to animals that were seronegative at the beginning of the experiment and became seropositive to BLV at 60 dpv

Groups BLV+ and BLV- remained seropositive and seronegative respectively, whereas the SC group seroconverted during the study between day 15 and 150 of the experiment (Fig. 1). Any animal showed leukocytosis or lymphocytosis at the beginning of the experiment, which was confirmed by hemogram in PBMC.

### Total FMDV antibodies

**Fig. 2:**
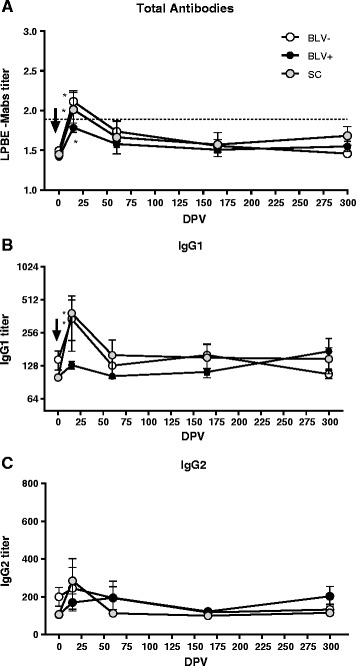
Kinetics of antibody titers against A24/Cruzeiro strain in BLV seronegative (BLV-; *n* = 10; white circles), BLV seropositive (BLV+; *n* = 20; black circles) and seroconverted groups (SC; *n* = 5; gray circles). Mean titers standard errors (SEM) are depicted. A) Total antibody measured by LPBE. **a** titer of 1.90 (dotted line) is considered to be related to an EPP 75 % for A24/Cruzeiro strain. **b** IgG1 and **c** IgG2 measured by ELISA. All animals received one dose of a commercial bivalent FMD vaccine at 0 dpv. Titers against A24/Cruzeiro were significantly higher in all groups at 15 dpv compared to 0 dpv (*p* < 0.01)

**Fig. 3:**
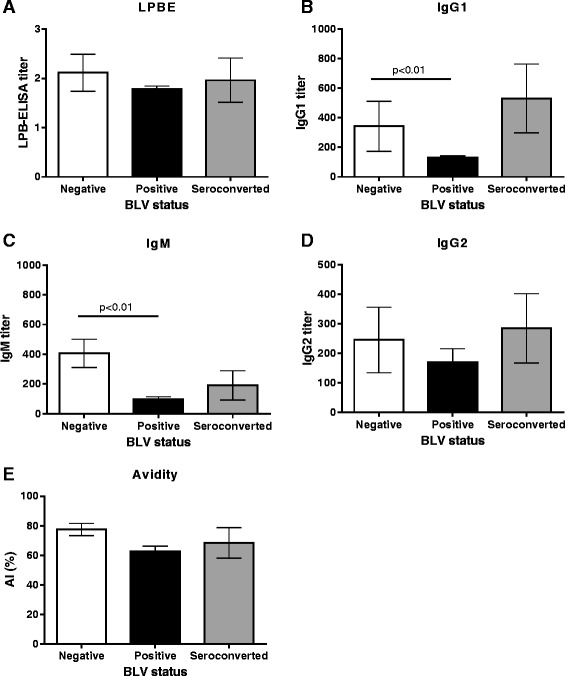
Comparison of LPBE (**a**), isotypes (IgG1, (**b**) IgM, (**c**) and IgG2, (**d**) and avidity index (**e**) against A24/Cruzeiro strain for (BLV-; *n* = 10; white columns), BLV seropositive (BLV+; *n* = 20; black columns) and seroconverted groups (SC; *n* = 5; gray columns) at day 15 post vaccination. Mean titers ± standard errors (SEM) are depicted. All animals received one dose of a commercial bivalent FMD vaccine at 0 dpv. IgM and IgG1 against A24/Cruzeiro were significantly higher in BLV – compared to BLV + at 15 dpv (*p* < 0.01, Mann-Whitney test)

LPBE kinetics curves were similar between the three groups up to day 300 of the assay. Total antibody titers increased after vaccination in all the animals and this difference was significant at 15 dpv for each group compared to titers measured at 0 dpv (*p* < 0,01). After that, the titers decreased and remained at low levels up to the end of the experiment. Antibody levels fell below the EPP 75 %- protective levels for FMD after 60 dpv (Maradei, et al. [19]) (Fig. 2a). Total FMD LPBE titers 15 dpv were not significantly different between groups (*p* > 0.05), although titers were higher in the BLV-compared with BLV+ group (Fig. 3a).

Avidity indexes kinetic curves were comparable between the groups. Antibody avidity was boosted 15 dpv, and the tendency seemed to be higher for BLV-animals, however, though differences were not significant compared to BLV+ animals (Fig. 3e).

The SC group behaved as the BLV-animals, both for total antibodies and avidity.

### Isotype responses

We then studied the isotype composition of the induced antibodies, to verify the primary (IgM) response and the switch towards IgG isotypes, indicative of T-cell collaboration. IgM, IgG1 and IgG2 titer against A24/Cruzeiro strain were measured in serum samples from the different groups along time.

An increase in IgM anti-FMDV serum titers was observed at 15 dpv in all groups, being significantly higher in BLV negative animals (Fig. 3c). IgG1 titers were also higher for BLV negative compared to BLV positive animals at 15 dpv (*p* < 0,01), with a mean titer of 287,1 y 127,8 respectively (Fig. 3b). At longer time-points, however, no significant differences were detected (Fig. 2b). IgG2 titers and kinetics were similar in all groups (Fig. 2c). Higher antibody titers were observed at 15 dpv in BLV negative animals, with a mean titer of 229.3 compared with 174.6 of BLV positive animals (Fig. 3d), although this differences were not significant (*p* > 0,05). In all cases, the SC group behaved as the BLV-animals in terms of isotype responses.

## Discussion

FMD has global consequences, costing an estimated USD $6–$21 billion each year in prevention expenditures and agricultural damage [9]. A significant portion of this cost is shouldered by the world’s poorest countries, which experience major economic losses from trade restrictions [21]. Uruguay, a small country where the main economy comes from agriculture and depends heavily on the export of products of animal origin, was free from FMD without vaccination until 2000. Later, after clinical signs manifestations in cattle, government turned to compulsory vaccination in all the country. Uruguay is actually classified as free from FMD with vaccination and invests millions of dollars to assure the maintenance of the FMD-free status granted by the World Organization for Animal Health.

On the other hand, BLV is another main virosis of dairy cattle, produces chronic infections with high percentage of asymptomatic animals. BLV infection reduces expression of type 1 cytokines from CD4+ T lymphocytes, and cytokine profiles from all peripheral blood mononuclear cell populations, suggesting that both type I and II cytokines are altered with increases in IL10 and IL4, and decreases in IL2, IL12, and IFN-γ [5, 22]. The progression of BLV is also known to disrupt the homeostasis of lymphocyte proliferation and cell death, in both B-cells and T-cells [1, 3, 4, 6].

Here we evaluated if the application of a bivalent FMD vaccine in BLV infected animals modify the humoral responses against A24/Cruzeiro strain included in the FMD vaccine. The study was designed to assess the FMD responses to match the practical approach applied in the field, where the probability of heifers to be infected with BLV at the moment of vaccination is very high, mainly in Uruguay, where the seroprevalence may reach 77 % in dairy cattle [23].

FMD immune response to vaccination is currently assessed using Liquid phase blocking ELISA (LPBE), and there are published curves relating LPBE titers with protection. An EPP of 75 % has been estimated to correspond to LPBE titers equal to 1.90 for A24/Cruzeiro strain [19, 20]. The animals included in this study presented low levels of antibody titers between 1.4 and 1.5 for A24/Cruzeiro at Day 0 of assay. This basal response could be associated to maternal antibodies transferred by colostrum because animals used were from 6 months age [24]. Even though, this response would not be interfering with oil emulsified vaccine applied in this assay [25].

After vaccination, every animal seroconverted at 15 dpv, with an average titer of 1.76 and 2.12 in BLV+ and BLV–, respectively. Although a greater response in non-infected heifers with titers higher than 1.90 (EPP of 75 %) was observed, this difference was not significant between groups (*p* > 0.05). After this period, a sharp decrease in antibody titers in all groups was observed at day 60, up to levels below 75 % of EPP and then remained low until the end of the experiment (Fig. 2). Based on these results and independently from BLV status of heifers, the single dose vaccination applied in this experiment was not enough to maintain animals with acceptable levels of protective antibody titers against FMD A24/Cruzeiro strain. Although an approved commercial vaccine was used and applied according to recommendations of the Uruguayan animal health authorities, the quality of this vaccine at the exact time it was applied could not be assessed by our working group, in terms of antigen payload and integrity of 140S particles, known to be essential for protection [26]. Thus, the data analyzed here correspond to the effect of a vaccine that elicits a short coverage of circulating antibodies within protective levels.

Analysis of the isotypes of the response induced against A24/Cruzeiro revealed that IgM, IgG1 and IgG2 titers increased in both BLV-and BLV+ heifers following FMD immunization, although IgM and IgG1 titers were higher in the BLV negative heifers. Levels of IgG2 can explain why the difference in antibody titers was only marginally significant when total antibodies were measured. On the other hand, while we found that the avidity index was lower in seropositive animals than that seronegative, these differences were not significant (Fig. 3e). The avidity index and IgM titers were measured only 15 dpv because these decreases rapidly according to previous reports from some researchers from our group (Lavoria et al. [12]).

It is important to note that peak titers of both IgM and IgG1 may not reflect, however, the true course of the kinetics of the immune response, as many time points are missing. Higher IgM titers have been reported to take place about 7 to 10 dpv while IgG1 peak may be expected at 15–20 dpv [15, 18]. Thus, we cannot rule out if we had either a typical primary-secondary response, with a first boost of IgM followed by a delayed increase of IgG; or if a T-independent response was induced in both cases, though with lower magnitude in BLV-infected animals.

We hypothesize that, if we consider the first scenario, the immune-modulation exerted on the T lymphocytes in BLV-infected animals may account for the lower magnitude of the IgG1 anti FMDV-response. Alternations in cytokine expression have been shown to be correlated with disease progression in chronic retroviral infections, suggesting that cytokine imbalances may contribute to disease progressions [2]. Pyeon et al. [27] found that the production of Th1 cytokine was promoted in the first stage of the BLV infection (serologically positive without persistent lymphocytosis) than that in cattle with progressed disease stages with persistent lymphocytosis. It has also been shown that IgG1 expression is positively regulated by IL-4 and IgG2 expression is positively regulated by IFN-gamma [28]. So, considering that the animals used in our study were young heifers that didn’t have persistent lymphocytosis at the beginning of the experiment and were probably in the early phase of infection (Th1 profile), we think that may have influenced immunoglobulin subclass switch. Further experiments are needed to support this hypothesis.

Animals that were infected with BLV between 15 to 60 dpv (“BLV seroconverted” group), did not show any difference with respect to BLV negative animals, indicating that only a pre-existing BLV infection modified the isotypes of antibodies against FMD induced by vaccination. This study is another contribution to the effect that BLV may have on immunization with commonly used vaccines. In this regard, Erskine et al. [1] also found differences on titer of BLV antibodies in infected cows when they were vaccinated against J5 *E. coli*.

## Conclusions

Our data suggests that BLV infection in dairy cattle may modify the profile of antibody response to immunization against Foot-and-mouth disease. IgM and IgG1 titers were significantly lower in heifers infected with BLV at the 15 dpv (*p* < 0.01). More studies using vaccines of different potency are needed to dissect the role of BLV infection on the efficacy of Foot-and-mouth disease primo-vaccination.

## Abbreviations

AI, Avidity Index; BLV, Bovine Leukemia Virus; dpv, days post vaccination; EPP, Expected protection percentage; FMD, Foot-and-mouth disease; FMDV, Foot-and-mouth disease virus; LPBE, Liquid phase blocking ELISA; MGAP, Ministerio de Ganadería Agricultura y Pesca; SC, Seroconverted; Th1, T helper 1 lymphocytes

## References

[1] Erskine RJ, Bartlett PC, Sabo KM, Sordillo LM (2011). Bovine leukemia virus infection in dairy cattle: effect on serological response to immunization against J5 Escherichia coli bacterin. Vet Med Int.

[2] Kabeya H, Ohashi K, Onuma M (2001). Host immune responses in the course of bovine leukemia virus infection. J Vet Med Sci.

[3] Sordillo LM, Hicks CR, Pighetti MG (1994). Altered interleukin-2 production by lymphocyte populations from bovine leukemia virus-infected cattle. Proc Soc Exp Biol Med.

[4] Stone DM, Hof AJ, Davis WC (1995). Up-regulation of IL-2 receptor α and MHC class II expression on lymphocyte subpopulations from bovine leukemia virus infected lymphocytotic cows. Vet Immunol Immunopathol.

[5] Konnai S, Usui T, Ohashi K, Onuma M (2003). The rapid quantitative analysis of bovine cytokine genes by real-time RTPCR. Vet Microbiol.

[6] Tyler JW, Cullor JS, Dellinger JD (1990). Cross-reactive affinity purification of immunoglobulin recognizing common gram-negative bacterial core antigens. J Immunol Methods.

[7] Florins A, Boxus M, Vandermeers F, Verlaeten O, Bouzar AB, Defoiche J (2008). Emphasis on cell turnover in two hosts infected by bovine leukemia virus: a rationale for host susceptibility to disease. Vet Immunol Immunopathol.

[8] Archambault D, Morin G, Elazhary MA (1989). Possible impairment of rotavirus immune response in cattle infected with BLV. Vet Rec.

[9] Smith MT, Bennett AM, Grubman MJ, Bundy BC (2014). Foot-and-mouth disease: technical and political challenges to eradication. Vaccine.

[10] Doel TR (2003). FMD vaccines. Virus Res.

[11] Capozzo AV, Periolo OH, Robiolo B, Seki C, La Torre JL, Grigera PR (1997). Total and isotype humoral responses in cattle vaccinated with foot and mouth disease virus (FMDV) immunogen produced either in bovine tongue tissue or in BHK-21 cell suspension cultures. Vaccine.

[12] Lavoria MA, Di-Giacomo S, Bucafusco D, Franco-Mahecha OL, Perez-Filgueira DM, Capozzo AV (2012). Avidity and subtyping of specific antibodies applied to the indirect assessment of heterologous protection against foot-and-mouth disease virus in cattle. Vaccine.

[13] Brito BP, Rodriguez LL, Hammond JM, Pinto J, Perez AM (2015). Review of the Global distribution of foot-and-mouth disease virus from 2007 to 2014. Transbound Emerg Dis.

[14] Pega J, Di Giacomo S, Bucafusco D, Schammas JM, Malacari D, Barrionuevo F (2015). Systemic foot-and-mouth disease vaccination in cattle promotes specific antibody-secreting cells at the respiratory tract and triggers local anamnestic responses upon aerosol infection. J Virol.

[15] Pega J, Bucafusco D, Di Giacomo S, Schammas JM, Malacari D, Capozzo AV (2013). Early adaptive immune responses in the respiratory tract of foot-and-mouth disease virus-infected cattle. J Virol.

[16] Marshak RR (1968). Criteria for the determination of the normal and leukotic state in cattle. J Natl Cancer Inst.

[17] Ministerio de Ganadería agricultura y Pesca (MGAP). Legislación Sanitaria Animal. Available at: http://www.mgap.gub.uy/portal/page.aspx?2,dgsg,dgsg-legislacion-sanitaria,O,es,0. Accessed 08 Dec 2015.

[18] Bucafusco D, Di Giacomo S, Pega J, Juncos MS, Schammas JM, Perez-Filgueira M (2014). Influence of antibodies transferred by colostrum in the immune responses of calves to current foot-and-mouth disease vaccines. Vaccine.

[19] Maradei E, La Torre J, Robiolo B, Esteves J, Seki C, Pedemonte A (2008). Updating of the correlation between lpELISA titers and protection from virus challenge for the assessment of the potency of polyvalent aphtovirus vaccines in Argentina. Vaccine.

[20] Robiolo B, La Torre J, Duffy S, Leon E, Seki C, Torres A (2010). Quantitative single serum-dilution liquid phase competitive blocking ELISA for the assessment of herd immunity and expected protection against foot-and-mouth disease virus in vaccinated cattle. J Virol Methods.

[21] Trotta M, Lahore J, Cardoso N, Melucci O, Catena M (2015). Simultaneous immunization of cattle with foot-and-mouth disease (FMD) and live anthrax vaccines do not interfere with FMD booster responses. Trials Vaccinol.

[22] Amills M, Norimine J, Olmstead CA, Lewin HA. Cytokine mRNA expression in B cells from bovine leukemia virus-infected cattle with persistent lymphocytosis. Cytokine. 2004;28(1):25–8. 10.1016/j.cyto.2004.06.004 15341922

[23] Furtado A, Rosadilla D, Franco G, Piaggio J, Puentes R (2013). Leucosis Bovina Enzoótica en cuencas lecheras de productores familiares del Uruguay. Veterinaria.

[24] World Organization for Animal Health (OIE) (2014). Foot and mouth disease. Terrestrial animal health code.

[25] Patil PK, Sajjanar CM, Natarajan C, Bayry J (2014). Neutralizing antibody responses to foot-and-mouth disease quadrivalent (type O, A, C and Asia 1) vaccines in growing calves with pre-existing maternal antibodies. Vet Microbiol.

[26] Bucafusco D, Di Giacomo S, Pega J, Schammas JM, Cardoso N, Capozzo AV (2015). Foot-and-mouth disease vaccination induces cross-reactive IFN-γ responses in cattle that are dependent on the integrity of the 140S particles. Virology.

[27] Pyeon D, Splitter GA (1998). Interleukin-12 p40 mRNA expression in bovine leukemia virus-infected animals: increase in alymphocytosis but decrease in persistent lymphocytosis. J Virol.

[28] Estes DM, Brown WC (2002). Type 1 and type 2 responses in regulation of Ig isotype expression in cattle. Vet Immunol Immunopathol.

